# Construction and Simulation of Music Style Prediction Model under Improved Sparse Neural Network

**DOI:** 10.1155/2022/6268224

**Published:** 2022-04-08

**Authors:** Junfang Wu, Junbiao Lu

**Affiliations:** ^1^School of Modern Service, Jining Normal University, Wulanchabu, Ulanqab 012000, China; ^2^School of Foreign Language, Jining Normal University, Inner Mongolia 012000, China

## Abstract

This paper designs and implements a music style prediction system using an improved sparse neural network, aiming to provide users with personalized music lists that match their interests. This paper firstly introduces how to combine the restricted Boltzmann machine model and recommendation algorithm and proposes a method to extract data features—setting a threshold to extract data features, and then, based on this, this paper introduces an improved *K*-Item RBM by weighted fusion of RBM recommendation algorithm and Item's recommendation algorithm. Finally, the algorithm model is trained and predicted by the extracted features, and the experimental comparison analysis shows that the *K*-Item RBM algorithm can reduce the error between the predicted data and the real data and improve the performance of the recommendation system; in addition, to improve the accuracy of the recommendation, this paper introduces an improved CNN-CF neural network recommendation algorithm, which uses a convolutional neural network (CNN) to extract. The algorithm uses a convolutional neural network (CNN) to extract text features from the dataset, then trains the algorithm model, and finally makes personalized recommendations to users. The system can crawl user and music data and complete preprocessing of data such as deduplication, word separation, and keyword extraction. In this paper, we define the prediction evaluation criteria with the evaluation index *F* as the core and compare and analyse the prediction effect of four models longitudinally. The experimental results show that the music style prediction model based on the improved sparse neural network has a higher evaluation index value F and better prediction performance than the two-time series prediction models; compared with the general sparse neural network music style prediction model, the improved sparse neural network music style prediction model has an increased evaluation index value F for prediction ability, and the overall prediction effect is better and the prediction ability is significantly improved. The system can judge the appropriate recommendation algorithm according to the actual situation of the user and music data information and realize the continuously personalized music list recommendation for users to meet their music needs.

## 1. Introduction

We are now in the golden age of rapid development of IT-related industries such as the Internet, cloud computing, big data, and mobile technology, and the amount of data generated from them is growing saturated year by year. However, due to the huge scale of user data storage and the existence of data redundancy and cumbersome problems, the use of data by ordinary users is generally low, which leads to the problem that service providers cannot accurately capture the differences in the use of individual users. Therefore, it has become a common goal for scholars to obtain valuable data from the troubled and complicated data ocean and provide better services to users [1]. Currently, there are two main ways to obtain information, namely, active information retrieval and passive recommendation systems. Among them, although the active information retrieval method can solve part of people's needs to a certain extent, the disadvantages of incomprehensive, impersonal, and diverse retrieval are exposed because users have different needs for data at different times and different purposes. Therefore, passive recommendation systems came into being along with such special conditions. The core of algorithmic art is digital technology, and the core of digital technology lies in the concept of digital characters to explain reality and study it through logical thinking. When many elements of music creation are digitized, the creator needs to change the original concept of creation based on perceptual thinking and then use more rational thinking, mathematical thinking, and computer language to solve specific problems in the creation process [2]. The essence of creation by digital technology lies in the statistics of data categories, data distribution, and data probability of different elements to form a mutual mapping between different data types, and the realization of the mapping needs to be realized through the process of data preprocessing, statistical classification, data normalization, and medicalization. In this process, the creator needs to think logically to deconstruct the content of the music data and to think logically about how to deal with the parameters that constitute the data content; on the other hand, he needs to clarify how to form new parameters for the data and build newly generated content, digital as a technical carrier provides more possibilities for artistic creation [3].

At the same time, Internet music users have put forward higher intelligent requirements and personalized needs for music platform usage experience, and how to better utilize various Internet music data for music users has become a popular research point today. In the Internet environment, many songs emerge every year, and Internet music platforms are popping up all over the place [4]. Whoever gains an advantage in music technology will occupy the market first. From the user's point of view, predicting music trends can let people intuitively understand the current popular singers in the music field and get the latest and recent development trends; from the enterprise's point of view, it can effectively predict the future dark horses in the singing world and make it easier to select singers worthy of cooperation, which is very helpful for the future development of the enterprise; from the market's point of view, knowing the precise music trends in advance [5]. From the perspective of the market, knowing the accurate music trend in advance will give you a head start in occupying the market share; from the perspective of the singers, if you know the trend of music popularity in advance, you can plan your development route earlier, understand the market demand earlier, and occupy a place in the singing world as soon as possible, which is very important guidance for the singers who are confused and harbouring dreams.

With the rapid development of Internet technology, while it brings a lot of convenience to users and meets their needs, it also brings the problem of information overload. How to quickly find the information of interest from the huge information has become very important, so personalized recommendation has become more popular. E-commerce platforms usually use the user's usual purchase records, portals usually based on the user's browsing news category, entertainment industry by analysing the user's viewing type of movies and other historical behaviour data to explore the user's interests and recommend relevant information to them. Recommendation algorithms are usually classified according to user dimensions, item dimensions, or deep learning models. Although the traditional collaborative filtering recommendation algorithm has been widely used, the algorithm still suffers from low recommendation accuracy and cold start problem of new items. This paper aims to improve the accuracy of the recommendation algorithm using an improved sparse neural network model.

## 2. Related Works

Currently, there are various applications of data mining in the field of information recommendation. According to the existing literature, recommendation algorithms are broadly classified into the following categories based on different recommendation strategies: Content-based Recommendation, Collaborative Filtering Based Recommendation, and Hybrid Recommendation. For better prediction performance, compared with the general sparse neural network music style prediction model, the improved sparse neural network music style prediction model has an increase in the F value of the evaluation index value of the prediction ability, the overall prediction effect is better, and the prediction ability is significantly improved. Researchers created the first recommendation system in history, Group Lens Recommendation System, which mainly recommended Usenet news [6], whose main technique was to use user ratings of movies to build a recommendation system by combining collaborative filtering and association rules. Amazon, which was the world's largest online bookstore at that time, used the Plug service to recommend some product listings to users by recording the transaction information between users and products, thus increasing the sales of products. Recommendation algorithms have evolved in the last decade or so. Recess conference has become one of the major ACM conferences [7]. Moreover, many excellent papers on recommendation systems have been included in ICDM, ECML, and other important conferences every year. In addition, in the academic theory, we have studied the selection method of feature weights and analysed the accuracy of recommendation algorithms; we have proposed a hybrid algorithm combining content-based recommendation algorithm and linguistic model, which collects information about users' description of interests and trains the algorithm model to realize personalized recommendation.

The accuracy of music style prediction has a significant impact not only on users' experience but also on the economic benefits of enterprises and singers [8]. In recent years, Internet enterprises have invested a lot of money and workforce in continuous research, and academics are also interested in the research of music style prediction and making it a research focus [9]. At present, after years of development, Internet music platforms now have a huge amount of song library resources and user behaviour records. Music style prediction can be analysed and predicted by using various historical data (e.g., based on users' preferences). Music style prediction can be achieved by using prediction models such as time series and regression. In 2017, by statistically analysing the behaviour records of users playing, downloading, and collecting songs generated by electronic music platforms, we combined quadratic exponential smoothing method and BP neural network model to predict the playback of singers' songs, and we developed and tested a music style prediction system based on BP neural network algorithm [10]. We analysed the data of monthly, weekly, and daily golden song charts, which symbolize the trend of popular songs in music platforms, and combined the two algorithms of support vector machine (SVM) and artificial neural network (ANN) linearly to build the music style prediction model using the combined model, and the combined model improved the prediction performance [11]. The historical data of music and listeners were analysed in a big data environment to achieve a more accurate prediction of music style prediction. In this paper, we will further explore and innovate the music style prediction problem based on the existing research [12].

With the rapid development of information society, the use of recommendation systems in daily life is becoming increasingly comprehensive. Among them, although the active information retrieval method can meet some people's needs to a certain extent, because users have different needs for data in different periods and different purposes, the disadvantages of incomplete, unindividual, and undiversified retrieval are also exposed. Although the above recommendation algorithms have been widely used in various fields of research, there are still some important problems in their actual use, so the research still needs to continue to explore.

## 3. Construction of the Music Style Prediction Model Based on the Improved Sparse Neural Network

### 3.1. Improved Sparse Neural Network Model Design

Transfer functions are an important component of the FCM and are used to map unbounded weights monotonically to a normalized range. The results of the study show that the Sigmoid function has a greater advantage over the other functions [13]. According to the comparative study, the Sigmoid function is suitable for both quantitative and qualitative analysis and is generally superior to the other functions. However, the Sigmoid function still has some disadvantages. For example, the inherent characteristics of the Sigmoid function make it easy to reach saturation; i.e., it will be at its maximum after a few iterations, which will lead to poor modelling ability of the FCM. Meanwhile, the existing literature does not investigate the ability of different transfer functions to model complex systems and handle pattern classification problems. In this chapter, RCGA is used as the FCM learning method to investigate the performance of the proposed wavelet transfer function. In learning FCMs, each chromosome represents a candidate FCM and consists of *N* × *N* genes, each with values in the range [−1, 1]. The chromosomes are defined as

(1) $$\begin{array}{c} W=\left[w_{11}^{2},w_{12}^{2},w_{13}^{2},w_{14}^{2},...,w_{1n}^{2}\right]. \end{array}$$

The GRN reconstruction problem is an important application of FCM. This section first presents the DREAM3 and DREAM4 datasets used in this paper. The DREAM time series presents variations of different networks with topologies based on real networks. Its dynamical properties are generated by continuous differential equations with noise and perturbations. DREAM provides networks of 10, 50, and 100 genes, with 5 different networks in each set [14]. We use the last 11 time points in each time series since the previous time points were collected under perturbation. To evaluate the performance of the FCM learning algorithm, the following performance metrics will be used in this paper:

(2) $$\begin{array}{c} \mathrm{Data\_Error}=\frac{1}{NST+NS}\sum_{i=1}^{N}\sum_{k=1}^{S}\sum_{t=1}^{T}{\left(C_{i}^{k}\left(t\right)+C_{i}^{k}{\left(t\right)}^{2}\right)}^{2}, \end{array}$$

where *T* is the length of the data sequence, S is the number of response sequences, and *C*_*i*_^*k*^(*t*) is the state value of node *i* at the *t* − th iteration in the *k* − th sequence. *C*_*i*_^*k*^(*t*)^2^ is the state value of node *i* at the *t* − th iteration in the *k* − th sequence, which is generated by the learned FCM.

**Figure 1:**
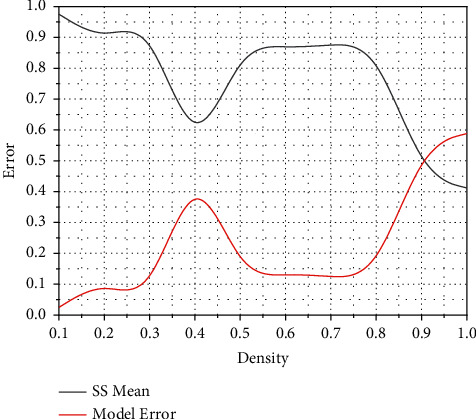
SS_Mean and Model_Error about density.

For different FCMs, when the density increases, *SS*_*Mean* decreases and *Mo*  *de*  *l*_*Error* increases. There are two reasons for the decrease. First, for dense FCMs, the sparsity of W is poor, leading to higher prediction errors in LASSOFCM. Second, in dense FCMs, nodes are connected to multiple neighbours. The realization of the mapping needs to be done through data preprocessing, statistical classification, data normalization, medicalization, and other processes. That is, when there is enough data, LASSOFCM can learn dense FCM with high accuracy, as shown in Figure 1.

In practical applications, noise is often present in the time series, so it is necessary to examine LASSOFCM in a more realistic context [15]. In the case of no noise or small noise variance, e.g., *σ*=0.001 , full learning can be achieved by increasing the NM. For larger noise variances, e.g., *σ*=0.01 , sparse FCM can achieve higher accuracy, which indicates that LASSOFCM is robust to noise in time series.

(3) $$\begin{array}{c} \mathrm{sim}\left(x,y\right)=\frac{1}{1-d\left(x,y\right)},\\ d\left(x,y\right)=\sum_{i=1}^{n}{\left(x_{i}-y_{i}\right)}^{2}. \end{array}$$

**Figure 2:**
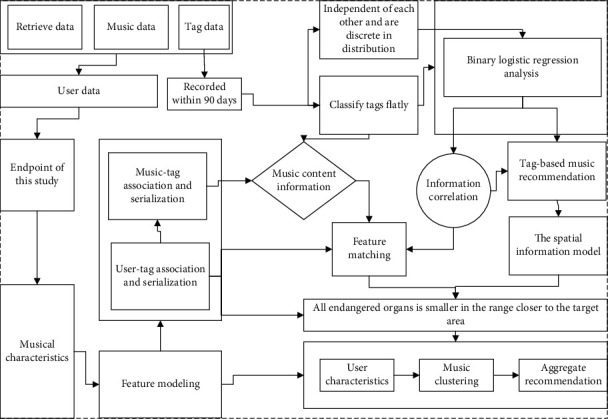
Flowchart of label-based collaborative filtering algorithm.

We collect the behavioural data such as time and number of times when users categorize and label music and vectorize them to reflect the relationship between users, music, tags, and cognitive order, to improve the accuracy of music recommendation as shown in Figure 2.

### 3.2. Music Style Prediction Model Construction

Music is the universal “language” of humankind. Any language has an orderly organization based on the temporal dimension, and its constituent elements include pitch and rhythm variations. At the same time, pitch and rhythm are two essential elements of melody, which are interconnected in an orderly manner. In most genres of applied music, the melody is the first element in the composition of music. How to make better use of various Internet music data to serve music users has also become a hot research point today. The core problem of this chapter is how to accurately represent these temporal and sequential characteristics of melodic and related musical data to the computer and input them into the neural network in the form of reasonable musical data representation. For the task to be implemented in this chapter, since the input MIDI file contains a large amount of data information, its data meaning symbolizes different note information, rhythmic information, chord information, performance information, etc. Furthermore, the input port of the neural network generally receives only real number information, and the network does not directly understand the meaning of the note information corresponding to the numbers in the music [16]. Therefore, this study requires the use of code to convert these note messages and chord messages into some numbers that can be easily accessed by the neural network as input. The common format of MIDI needs to be parsed, and the Fast Fourier Transform (FFT) algorithm is used as an aid to facilitate the accurate extraction of key data useful for subsequent sessions.

(4) $$\begin{array}{c} \rho_{x,y}=\frac{E\left[\left(x-\mu \right)\left(y-\mu \right)\right]}{\sigma_{x}\sigma_{y}}. \end{array}$$

In the MIDI file header data module, the data in the MIDI file is divided into 3 data blocks at the first level, whose main control is the basic global information instructions such as tempo, beat number, key, and the clip start position of the piece. The data blocks consist of different block identifiers, block lengths, and formatting information. Based on the structural generality of MIDI data, the next step will be to analyse the valid music data information that needs to be extracted and matched with the MIDI data structure, so that it can be completely handed over to a prewritten computer program to extract the valid samples, thus saving the time cost of a lot of complicated manual operations and improving the efficiency while providing the most accurate training for the subsequent training. The global information of the dataset can be easily *normalized* for feature extraction. As shown in Table 1, the code is divided into 4 segments into units of 4 bytes, where the first character is the 4-byte ASCII code character “March,” which is used to identify whether the file is a standard MIDI file and to read the data subsequently. The next 4 bytes (00 00 0006) specify the number of bytes in the header description section, which occupies 6 bytes, so its only combination is “00 00 00 06.” So, in the preprocessing of MIDI data, you can check for the presence of nonstandard MIDI files by identifying the “MTrk” code field.

Fixed number of *K*-neighbourhoods (KNN): regardless of the “distance” of neighbours, only the nearest *K* are taken. For the calculation of isolated points, a fixed number of neighbouring nodes are taken, and when there are not enough similar points near the point, it is forced to get some points that may be less like the current point as neighbours, so it will affect the similarity between neighbours. Therefore, this method is not suitable for calculating isolated points, and in the above figure, when 5 points are taken, 1 and neighbour 5 are not very similar. Threshold Based Neighbourhoods: unlike the strategy of calculating a fixed number of *K* neighbours, this strategy is based on setting a threshold value to get neighbours, and the specific idea is to draw a circle with the current point as the centre and the distance *K* as the radius, and all points falling within the circle are considered as neighbours of the current point [17]. The number of neighbours obtained by this method is uncertain, but the similarity between neighbours will not deviate too much. From the perspective of the enterprise, it can effectively predict the dark horse in the future music scene, and it is more convenient to select singers worthy of cooperation, which is very helpful for the future development of the enterprise. The number of neighbours obtained by this method is uncertain, but the similarity between neighbours does not deviate too much. The neighbours of point 1 are calculated with the similarity threshold set to K, and the four points 2, 3, 4, and 7 can be obtained by calculation.

When recommending items to users, first find *K* neighbours according to the target user *u*'s preference for each item by one of the ways introduced above, *S*(*u*, *K*) denoted by extracting the items liked by *K* neighbouring users in *S* and removing the items already liked by the target user *u*. For each remaining candidate item marked by *i*, the target user *u*'s interest level in *i* is denoted by (5) to calculate

(5) $$\begin{array}{c} p\left(u,i\right)=\sum_{v\in S\left(u,K\right)\cap N\left(i\right)}w_{wv}*R_{vi}. \end{array}$$

By sorting the current user's interest in each candidate item, a ranked list of item recommendations can be obtained and presented to the current user.

**Figure 3:**
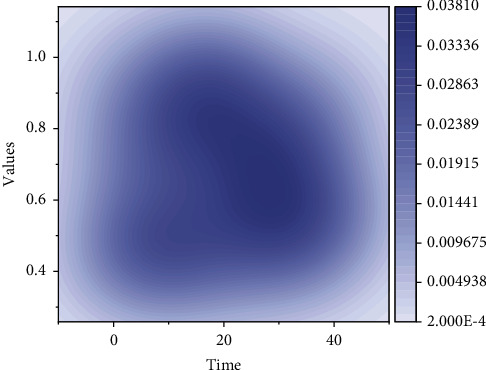
Singer's plays, downloads, and collections.

Based on ensuring the quality of the data, we visualize the distribution of the data through tools such as programming and plotting software and thus observe the data distribution. Distribution analysis can dig out the characteristics and types of data distribution. If the data are quantitative, frequency distribution tables and histograms can be created to visualize the distribution characteristics; if the data are qualitative, pie charts, bar charts, line graphs, etc. can be drawn to visualize the distribution status [18]. The comparison of two or more related indicators, such as the comparison in terms of the number of data, as well as the comparison of the level of prediction of the research algorithm and the accuracy of prediction, can also be based on the basic distribution of data for comparative analysis. This analysis method is particularly suitable for horizontal and vertical comparisons between indicators and comparative analysis of time series. For example, if the horizontal and vertical comparisons are made based on the number of downloads, collections, and plays of singers, they can be classified into stable singers and sudden increase singers. The specific classification method is the standard deviation of the singer's 183 days of plays > the set threshold, as shown in Figure 3.

Before the decision tree can be generated, the system needs to be run in the company for a period to generate a certain amount of data, including employee information on self and the project leader's evaluation of the employee and the final appraisal score, from which a decision tree is generated. A decision tree is generated from this data set.

The decision tree is used to analyse the attributes of interest in the performance appraisal and to help employees with their self-management and planning. After testing the decision tree, if the accuracy is satisfactory, the results of the decision tree can be used for performance appraisal scoring.

The system automatically generates performance appraisal scores based on the decision tree and the information provided by the employee.

In the experimental process, even the best models will not give good results if there is dirty data, and often the obtained data will have missing values, duplicate values, etc., which require data preprocessing before use. The data features obtained by One-Hot encoding of music data are highly sparse [19]. However, the sigmoid function still has some drawbacks. For example, due to the inherent characteristics of the sigmoid function, it is easy to reach a saturation state; that is, it will be at a maximum value after a few iterations, which will lead to poor modelling ability of FCM [20]. In the data example, there are 3-bit, 9-bit, and 3-bit possible values for user's operation behaviour, song language, and artist's gender, respectively, so 3-bit, 9-bit, and 3 bit-binary feature values are used, respectively. These category features are encoded by One-Hot resulting in sparse and high-dimensional music data. For example, the user's collection date has 183 categories, so data sparsity is inevitable.

## 4. Results and Analysis

### 4.1. Results of the Improved Sparse Neural Network Model

It is well known that traditional deep learning frameworks for pattern classification almost do not consider the relationships between input samples in the original data space. To consider the relationship between the input samples, we apply the following sample combination mechanism. When *K* = 1, the obtained new sample training set becomes the original sample training set, i.e., *X*(1)=*X*. For convenience, we will refer to it as the combination number, which takes a range of values max{*N*_*c*_, *c* ∈ *N*} ≥ *k* ≥ 1. Obviously, as the number of combinations increases, the number of new training samples obtained also increases exponentially.

**Figure 4:**
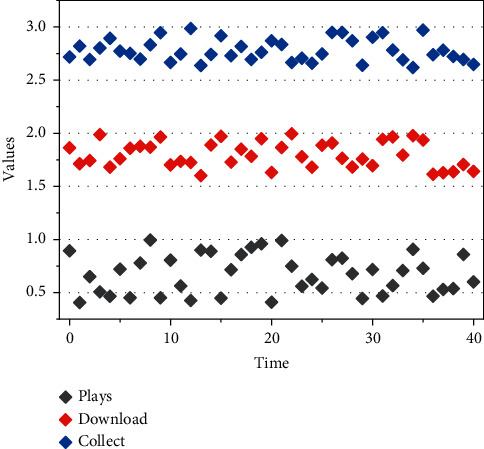
Performance trend of the 4SDCNN network.

Note that, in these experiments below, the effect on data preprocessing techniques has been ignored, since we mainly focus on the generalization ability of the network. We consider the following two scenarios: Scenario I is assumed and the number of channels increases from 1 to 10; in Scenario II, when *S* = 2, for the number of channels varies from 1 to 25. Considering the randomness present in the modules in the SDCNN, our experiments are run 100 times and the average performance results obtained are shown in Figure 4.

**Figure 5:**
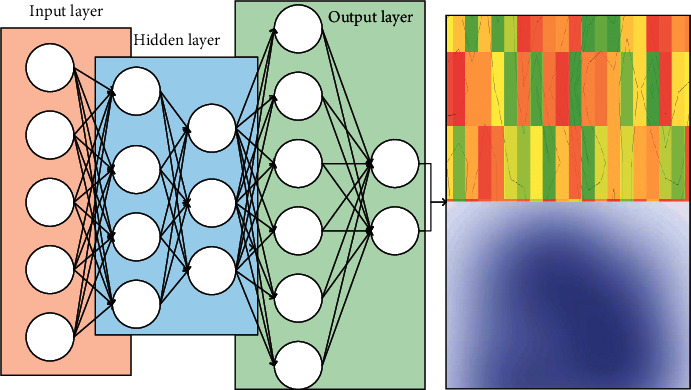
DSAE network structure diagram.

Along with the continuous research on AE, a series of network models such as sparse self-encoder, noise-reducing self-encoder, convolutional self-encoder, and drinkability self-encoder have emerged. Whether AE is considered as feature extraction or data dimensionality reduction algorithm, the hidden layer output is designed to form an efficient representation of the input. DREAM3 provides networks of 10, 50, and 100 genes, with 5 distinct networks per group. DREAM4 provides networks of 10 and 100 genes, also 5 different networks per group. Usually, for most application tasks, using a single hidden layer self-encoder alone is not sufficient to obtain a better feature representation. For pattern classification tasks, we can use deep neural networks with more hidden layers to obtain a better feature representation. Naturally, the AE is stacked layer by layer to obtain the following deep-stacked self-coding network, whose network structure is shown in Figure 5.

The DSAE network of Figure 5 has two components: one is the first three hidden layers for layer-by-layer feature learning, and the other is the last hidden layer to the output for the classifier design. DSAE initializes the parameters in the network by pretraining with layer-by-layer unsupervised learning and then fine-tunes this network by fine-tuning it end-to-end as a whole to improve the convergence speed and acquire relatively abstract features. Specifically, each self-encoder corresponds to a hidden layer, where the first layer is the original training data transformed into the hidden layer as input, and its parameters are determined by minimizing the training reconstruction error of the self-encoder; then the output of the hidden layer is used as input to the next layer, and the parameters of this layer continue to be determined by minimizing the training reconstruction error of the self-encoder. And so on, the hidden layer of the previous layer is the input of the next layer, and the parameters of each layer are determined by minimizing the reconstruction error, which is a very important application of stacked self-encoders for building deep neural networks.

**Figure 6:**
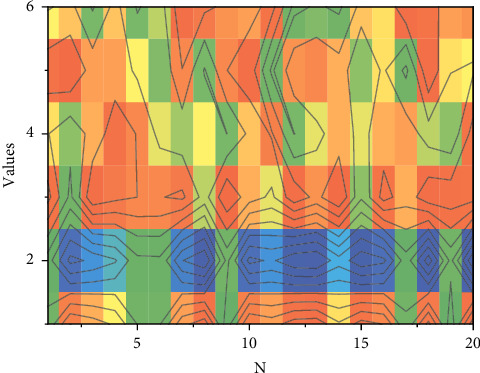
Test results of the improved sparse neural network model.

The results show that the length of the data sequence has a significant impact on the performance of MOEANet and that most connections can be recognized even for small values of *k*, as can be derived from the high values of AUROC and AUPR. However, we observe that RE decreases rapidly with increasing NM. When NM exceeds a certain value, RE is approximately 0, which indicates that all edge weights have been successfully predicted and no failed and redundant edges exist, even though the edge weights are random. This section also examines the ER and WS networks and observes that the data requirements can be slightly relaxed to achieve the same accuracy as compared to the BA and NW networks. In the case of no noise or very small noise variance, e.g., *σ*=0.0 or 0.05, a small amount of data relative to the network size *N* can guarantee a high reconstruction rate. For large noise variance, e.g., *σ*=0.3, the algorithm can still achieve a high reconstruction rate with a relatively large amount of data, which demonstrates the robustness of the method to noise in the time series. The effect of *k* on MOEANet is also discussed in this section. Experiments were performed on 100 nodes of FCM with densities of 20% and 40%, respectively. NM was increased from 0.1 to 3 in steps of 0.1, and each data point was obtained by averaging 30 independent runs. The experimental results show that, for larger values of *k*, MOEANet can guarantee the complete identification of all connections and weights. As K increases, a larger NM value than the sparse network is still required to obtain a higher reconstruction rate. The test results of the improved sparse neural network model are shown in Figure 6.

## 5. Music Style Prediction Model Simulation Experiment

We crawl NetEase Cloud Music comments from NetEase Cloud Music and store them in the text. Each page of comments under each song is stored in a file named R_SO_4_298101, where the number is the music id. params and unchecked are used to get all the comments under different pages, the initiator of the request is core.js, and the parameters params and unchecked are variables in the windows. The function contains 4 parameters; using Autoresponder in fiddler to download js to local output parameters, we found that only the first parameter is a variable, the next are constants, and the function calls the *d* function and *c* function to encrypt the parameters, so we can get the two parameters of the generation principle to get all the comments content.

**Figure 7:**
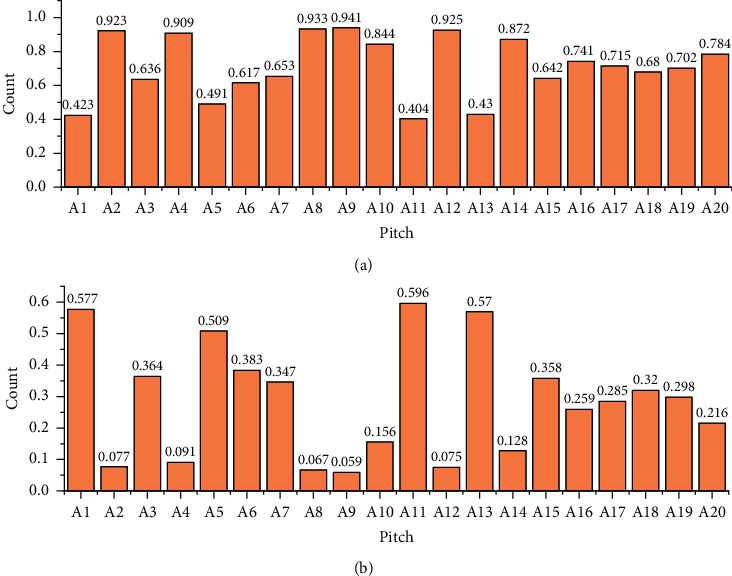
Histogram of notes before and after 7 key normalizations.

We present the statistics of the pitch counts before and after Key normalization of 20 random music files in the dataset concerning pitch type. However, there is a lack of thinking about the user's theme; correspondingly, the tag recommendation technology based on collaborative filtering mainly obtains the user's tagging behaviour data and infers music that is like the user's needs or compares it with the user's hobbies according to the data rules (similar users). The horizontal coordinates of the graph represent the Pitch types, and the vertical coordinates represent the count statistics of the corresponding Pitch types. The left histogram is before processing and the right histogram is after processing. The distribution of the counts corresponding to the note types can be seen, and good distribution is obtained. The notes before and after Key normalization are shown in Figure 7.

**Figure 8:**
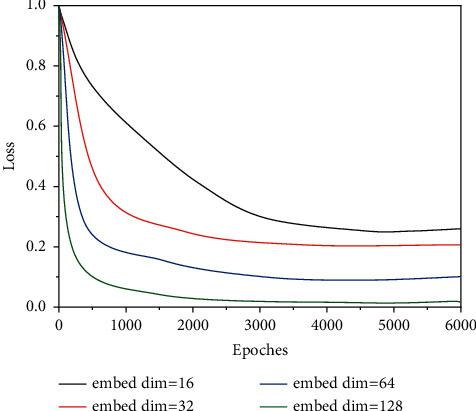
Simulation results of the music style prediction model.

The goal of the experiment is to train the user features and movie features for use in the recommendation. Once the above two features are obtained, the movie ratings can be fitted arbitrarily. In this section, the two features are fully concatenated as described in Figure 7, and then the obtained results are regressed against the real rating data, using the least square loss function MSE to optimize the loss. This is because recommendation is in essence a regression problem. However, tags are independent of each other and are discretely distributed. We cannot obtain what users think when they label or classify music, and we cannot directly obtain their label cognitive order. In this algorithm model, the main parameters affecting the experimental results are, embedding matrix dimension embed dim, learning rate, etc. Using the control variable method commonly used in scientific experiments, the following experiments are conducted by adjusting different parameters of the model parameters and the embedding matrix increases from small to large, and keeping the same number of training, learning rate, and the number of convolution kernels constant, we get the experimental results. As the dimension of the embedding matrix increases, the stability of the algorithm gradually decreases, and it is obvious from the first figure that the algorithm converges worst when embed dim = 128. Therefore, in this algorithm, the embed dim parameter is chosen as small as possible, but when the embed dim parameter is too small, the amount of the original data extracted will be too small, which will not represent the original data well. Therefore, among the above values, embed dim is chosen to be 16 more reasonable, as shown in Figure 8.

## 6. Conclusion

In this paper, we developed a crawler that can obtain massive music data and user data information from music stations such as MSD and Last. After completing the collection of data, we can extract music titles, artists, tags, and frequencies and the number of users tries and downloads from this information. In this paper, the problem of path selection in mental health prediction models is introduced. When the user is playing music, according to the user's own feelings about the music, they select the corresponding label in the music label category or complete their own labelling by creating their own label for the music; the user may have completely different feeling after listening to the same song repeatedly. The reason for this is that, in the analysis of mental health factors by means of the subject's underlying attribute information, the degree of correlation between each mental health factor and the subject's underlying information is different, and when predicting different mental health factors, it is necessary to choose the best path for the classification. The main purpose of this experiment is to demonstrate the difference in prediction accuracy between the FCTCP model and the CACS model for certain mental health factors. The performance of the TSCF algorithm was verified by dividing these data sets into test set training sets, and the algorithms were compared by the recall, accuracy, and F-value in a peer-reference experiment. The algorithms are compared in terms of recall, accuracy, and F-value to demonstrate that this music style prediction algorithm performs relatively well in practical applications [20].

## Figures and Tables

**Table 1:** ASCII character cross-reference table.

Binary	Decimal	Hex	ASCII characters
0100 1101	77	4D	M
0101 0100	84	54	T
0111 0010	114	72	r
0110 1001	107	6B	k

## Data Availability

The data used to support the findings of this study are available from the corresponding author upon request.
